# Spinal shock in a dog with steroid‐responsive meningitis‐arteritis extending to the brainstem

**DOI:** 10.1111/jsap.13806

**Published:** 2024-11-03

**Authors:** T. Liatis, A. Chardas, D. Cavalli‐Sforza, A. Skarbek, F. Llabres‐Diaz, S. De Decker

**Affiliations:** ^1^ Department of Clinical Sciences Royal Veterinary College Hatfield UK; ^2^ Department of Pathobiology and Population Sciences Royal Veterinary College Hatfield UK

A 3‐year‐old female entire cocker spaniel presented with acute progressive lethargy, pyrexia and tetraplegia. Neurological examination revealed tetraplegia with intact nociception, decreased withdrawal reflexes in all limbs, intact patellar reflexes, absent cutaneous trunci reflex, bilateral Horner syndrome, inconsistent menace response bilaterally, aphonia and diffuse spinal hyperaesthesia. Neuroanatomical localisation was to the C1‐5 spinal cord segments with spinal shock, diffuse spinal cord ± brainstem.

**FIG 1:**
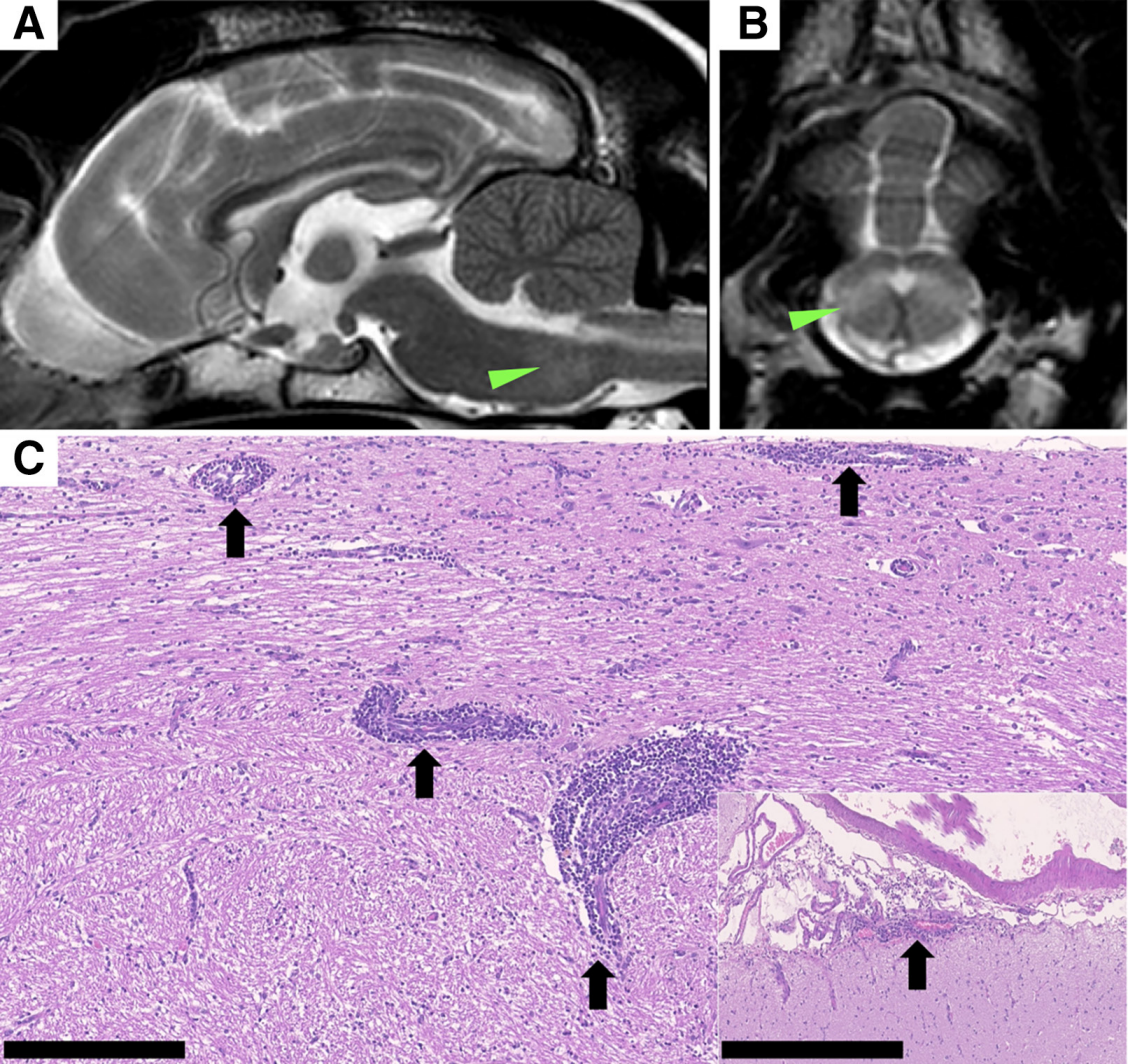
Magnetic resonance imaging of the brain of a dog with steroid‐responsive meningitis‐arteritis with intracerebral/intramedullary extension consisting of sagittal T2‐weighted (T2W) image of the brain (A) and transverse T2W image of the brain at the level of medulla oblongata (B). There is an extensive ill‐defined area of T2W hyperintensity relative to normal grey matter in the brainstem (arrowhead; A, B). Histopathology of the same dog including the brainstem and meningeal vasculature (inset) showing multifocal necrotising vasculitis with perivascular infiltrates of macrophages, lymphocytes, plasma cells and neutrophils (black arrows) (C; H&E stain; 250 μm scale bars).

Haematology revealed neutrophilia [14.21 × 10^9^/L, reference interval (RI): 3.00 to 11.50 × 10^9^/L]. Serum biochemistry revealed increased C‐reactive protein [81.9 mg/L, RI: <10 mg/L]. Thoracic and abdominal radiographs were unremarkable. Magnetic resonance imaging (MRI) (Intera 1.5 T, Philips Healthcare, Amsterdam, Netherlands) of head and neck demonstrated multifocal bilateral asymmetric ill‐defined intra‐axial mildly contrast‐enhancing T2W hyperintense lesions affecting the corpus striatum, periventricular region and brainstem (Fig 1), and a longitudinal poorly‐defined non‐contrast‐enhancing T2W dorsal intramedullary hyperintensity from C1 to T1 spinal cord. Cerebellomedullary cisternal cerebrospinal fluid (CSF) analysis revealed increased total proteins (2.96 g/L, RI: <0.25 g/L) and a marked neutrophilic pleocytosis (total nucleated cell count: 2455 cells/μL, RI: <5 cells/μL) with 68% non‐degenerate neutrophils, 20% monocytes, 7% lymphocytes, 5% macrophages and the presence of leukophagia. Differential diagnoses included infectious or immune‐mediated meningoencephalomyelitis or neoplasia. Euthanasia was elected. Post‐mortem examination revealed diffuse neutrophilic and histiocytic leptomeningitis and necrotizing fibrinoid thrombotic arteritis affecting the leptomeningeal arterioles in the cervical spinal cord and brainstem. Rarefaction, gliosis, necrosis and haemorrhages were present in the adjacent neuroparenchyma. Infectious agents were not visualised. A diagnosis of steroid‐responsive meningitis‐arteritis (SRMA) was made.

SRMA with intracranial/intramedullary extension is rare and should be considered in a young dog with MRI and CSF analysis consistent with neutrophilic meningoencephalomyelitis. Although experimentally spinal shock has been associated with brainstem transection in dogs, this is the first clinical case to describe spinal shock in a dog with brainstem and cervical spinal cord lesions.

